# Identification of six novel variants from nine Chinese families with hypophosphatemic rickets

**DOI:** 10.1186/s12920-022-01305-w

**Published:** 2022-07-16

**Authors:** Yixuan Cao, Yi You, Qiong Wang, Xiuzhi Ren, Shan Li, Lulu Li, Weibo Xia, Xin Guan, Tao Yang, Shiro Ikegawa, Zheng Wang, Xiuli Zhao

**Affiliations:** 1 Department of Medical Genetics, Institute of Basic Medical Sciences, Chinese Academy of Medical Sciences and School of Basic Medicine, Peking Union Medical College, Beijing, 100005 China; 2 The People’s Hospital of Wuqing District, Tianjin, 301700 China; 3 Department of Endocrinology, Key Laboratory of Endocrinology of the Ministry of Health, Peking Union Medical College Hospital, Chinese Academy of Medical Science and Peking Union Medical College, Beijing, 100730 China; 4 Laboratory for Bone and Joint Diseases, RIKEN Center for Integrative Medical Sciences (IMS), Tokyo, 108-8639 Japan

**Keywords:** Hypophosphatemic rickets, *PHEX*, *SLC34A3*, Variant, Genetic diagnosis

## Abstract

**Background:**

Hypophosphatemic rickets (HR) is a rare genetic disorder associated with renal phosphate wasting and characterized by bone defects. Inactivating mutations in the phosphate regulating endopeptidase homolog X‑linked gene (*PHEX*) account for most cases of HR. The aim of this study was to identify causative variants in nine unrelated Chinese families associated with HR, and to determine potential pathogenicity of the identified variants.

**Methods:**

Genomic DNA was isolated from the peripheral blood of HR patients and their healthy relatives, followed by next-generation sequencing and/or Sanger sequencing. In silico prediction combined with conservation analysis was performed to assess the effects of the variants, and 3D protein modeling was conducted to predict the functional effects on the encoded protein.

**Results:**

All HR patients recruited in this study displayed bone deformities and tooth agenesis, as well as reduced serum phosphate levels and elevated urine phosphate levels. Nine *PHEX* variants were identified in eight families, including four novel variants (c.1661_1726del, c.980A > G, c.1078A > T, and c.1017_1051dup). Of the nine identified *PHEX* variants, five caused a truncated protein, two caused an altered amino acid, and the other two were the canonical splicing variants. Novel variants c.1336G > A and c.1364 T > C in *SLC34A3* were also found in one family. Conservation analysis showed that all the amino acids corresponding to the missense variants were highly conserved. In silico analysis and 3D protein structure modeling confirmed the pathogenicity of these variants.

**Conclusions:**

This study identified four novel variants in *PHEX* and two novel variants in *SLC34A3* in a Chinese cohort with HR. Our findings highlight the dominant role of *PHEX* in HR, and expand the genotypic and phenotypic spectra of this disorder.

**Supplementary Information:**

The online version contains supplementary material available at 10.1186/s12920-022-01305-w.

## Background

Hypophosphatemic rickets (HR) is a group of metabolic bone diseases characterized by growth retardation, progressive bowing, abnormal bone and dentin formation, and short stature [1]. HR was first described as vitamin D-resistant rickets by Albright et al. in 1937, which differentiated it from general rickets caused by nutritional deficiency [2]. Although HR is not affected by the level of vitamin D, an excess of the hormone fibroblast growth factor 23 (FGF23) in circulation is believed to play a critical role in many HR cases. Current biochemical indicators of HR include elevated levels of FGF23 and serum alkaline phosphatase, as well as urinary phosphate excretion, and reduced levels of serum phosphate, and low to normal levels of serum calcium and serum 1,25(OH)2D [3, 4].

Up to now, there are 10 candidate genes contribute to congenital HR [3]. Most HR types are FGF23-dependent, including X-linked dominant HR (XLHR) (MIM 307800, caused by mutations in *PHEX*), autosomal dominant HR (ADHR) (MIM 193100, caused by mutations in *FGF23*), autosomal recessive HR (ARHR) type 1 (MIM 241520, caused by mutations in *DMP1*), and autosomal recessive HR (ARHR) type 2 (MIM 613312, caused by mutations in *ENPP1*). Meanwhile, FGF23-independent types of HR include HR with hypercalciuria (HHRH) (MIM 241530, caused by mutations in *SLC34A3*), HR with nephrolithiasis and osteoporosis type 1 (MIM 612286, caused by mutations in *SLC34A1*), HR with nephrolithiasis and osteoporosis type 2 (MIM 612287, caused by mutations in *SLC9A3R1*), HR with hyperparathyroidism (MIM 612089, caused by mutations in *KLOTHO*), X-linked HR (Dent syndrome) (MIM 300554, caused by mutations in *CLCN5*), and X-linked HR (Lowe syndrome) (MIM 309000, caused by mutations in *OCRL1*) [3, 5].

XLHR is the most common inherited HR type [6, 7] and accounts for more than 80% of all documented HR cases, with an incidence of 1 in 20,000 live births [8]. This disorder is caused by inactivating mutation in the *PHEX* gene. The *PHEX* gene, involved in the etiology of XLHR, encodes a membrane endopeptidase that is abundantly expressed in osteoblasts, odontoblasts, and chondrocytes [9, 10]. Levels of FGF23 is well known to be elevated in XLHR patients [3], as well as in a HR mouse model [11], and plays a key role in HR progression [12]. A study of Hyp mice, an animal model of XLHR caused by mutations in the *PHEX* gene [13], showed that FGF23 acted downstream of *PHEX* and that a *PHEX* mutation directly enhanced the secretion of FGF23 by osteoblasts, leading to reduced renal phosphate resorption and disrupted bone phenotypes [14]. Elevated FGF23 levels have been shown to inhibit the transcription of the genes *SLC34A1* and *SLC34A3*, encoding NaPi-IIa and NaPi-IIc, respectively [15]. In addition, suppression of NaPi-IIa and NaPi-IIc results in impaired renal phosphate reabsorption, and thus an accelerated manifestation of the HR phenotype [16].

Although different types of HR share common clinical manifestations, they have distinct causative factors and follow different patterns of inheritance. Therefore, genetic analysis of HR can aid precise diagnosis and direct therapeutic interventions. Prompted by the availability of next-generation sequencing, molecular diagnosis of HR has been performed in many populations including Europeans [17], Turkish [18], Americans [19], Chinese [20–22], and Japanese [23]. To date, 623 mutations in *PHEX* have been found to contribute to the development of XLHR (HGMD Professional, release 2020.04). In the present study, we recruited 17 HR patients from nine Chinese families to further investigate the pathogenic genes. Nine pathogenic variants in *PHEX*, and two variants in *SLC34A3* were identified, which included four novel variants in *PHEX* and two in *SLC34A3*. These findings expand the mutational spectra of *PHEX* and *SLC34A3*, and provide evidence of the dominant role of *PHEX* in HR development.

## Materials and methods

### Subjects

Nine unrelated Chinese families with HR were recruited in this study. In total, 56 individuals (27 male and 29 female) were included, 17 of whom had been diagnosed with HR (5 male and 12 female). Data on variables such as sex, age, height, weight, bone phenotypes, dental phenotypes, and available biochemical indicators were collected at their first visit. Height was converted to age- and sex-specific SDS Z-score based on the standard of the Chinese population [24]. Peripheral blood was collected from all probands and their available family members.

### DNA/RNA isolation

Genomic DNA was isolated from peripheral blood using the conventional proteinase K‐phenol‐chloroform method [25]. Total RNA was obtained using Trizol reagent (Invitrogen, CA, USA) from peripheral blood, in accordance with the manufacturer’s instructions. Subsequently, cDNA was prepared from 1 µg of RNA using PrimeScript RT reagent kit with gDNA eraser (TaKaRa).

### Sanger sequencing and genomic panel sequencing

Sanger sequencing was employed to screen the variants in *PHEX*. Primers designed for each exon are listed in Additional file 1: Table S1. DNA was amplified using a PCR system (TaKaRa). Sequencing was performed using Applied Biosystems 3739xl DNA analyser (Thermo Fisher Scientific, Waltham, MA, USA). For the probands with no variant identified in *PHEX*, customized panel sequencing including 184 skeleton-related genes (Additional file 1: Table S2) was conducted as previously described [25]. A total of 1–3 μg genomic DNA was used for panel sequencing. Briefly, DNA samples were sheared into 200 bp fragments using a Bioruptor NGS sonication device (Diagenode, Seraing, Belgium). After purification and library construction, hybridization reactions were performed, followed by sequencing performed on a HiSeq 2500 system (Illumina, San Diego, CA, USA). Sanger sequencing was used to verify the variants in the candidate genes. The sequencing results were analyzed using CodonCode Aligner (version 6.0.2.6; CodonCode, Centerville, MA, USA).

### Reverse-transcription PCR (RT-PCR) and amplicon sequencing

RT-PCR was carried out to determine the effects of the variants at the mRNA level. Primers were designed (Additional file 1: Table S1) via the online tool Primer 3 (http://primer3.ut.ee/), followed by examination using in silico PCR (http://genome.ucsc.edu/cgi-bin/hgPcr). The PCR products were separated on 2% agarose gel, and separated bands were isolated and processed for amplicon sequencing.

### Bioinformatic analysis

Conservation analysis was conducted via multiple alignments using Molecular Evolutionary Genetics Analysis version X (MEGA-X, https://www.megasoftware.net/). To predict the likelihood of mutation pathogenicity, Protein Variation Effect Analyzer version 1.1 (PROVEAN, http://provean.jcvi.org/seq_submit.php) and Polymorphism Phenotyping version 2 (PolyPhen-2, http://genetics.bwh.harvard.edu/pph2/) were employed. Human Splicing Finder version 3.1 (http://www.umd.be/HSF3/) was used to predict the effects of intronic variants on splicing. Protein modeling of PHEX and the protein encoded by the *PHEX* variant c.1661_1726del (p.Glu554_Gly575del) was performed using the online tool Phyre2 (http://www.sbg.bio.ic.ac.uk) [26]. The 3D structure of the PHEX protein was visualized by EZMOL (http://www.sbg.bio.ic.ac.uk/ezmol/). 3D models of wild-type proteins and the mutant proteins c.980A > G(p.Tyr327Cys) and c.1735G > A(p.Gly579Arg) were constructed using Swiss-Model (http://swissmodel.expasy.org) and residue analysis was conducted by PyMOL (http://www.pymol.org). The template used for PHEX protein modeling was based on Neprilysin (NEP, PBD: 5JMY).

## Results

### Clinical manifestations

**Fig. 1:**
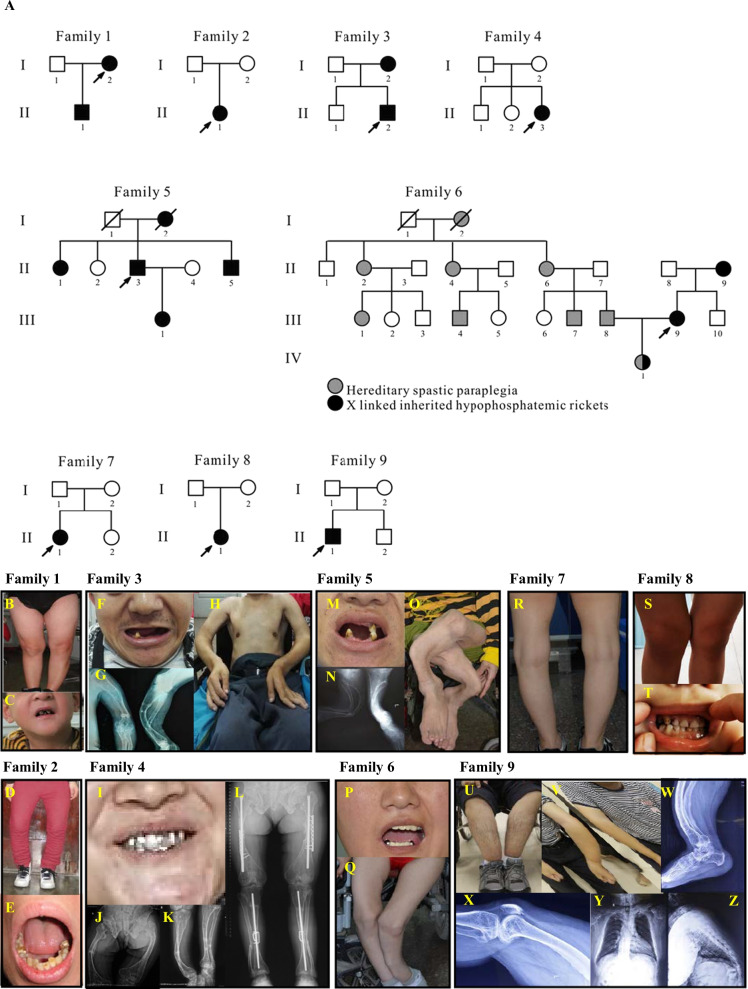
Clinical information and genetic background of the nine HR families.** A** Pedigrees of nine Chinese families with HR. Black symbols indicate individuals with HR, while open symbols represent unaffected individuals. Arrows indicate probands and slashes indicate deceased individuals. Squares represent males and circles represent females. In Family 6, gray symbols indicate affected individuals with hereditary spastic paraplegia and black symbols indicate affected individuals with HR. **B–Z** Clinical manifestations of HR patients

In this study, nine Chinese HR families were recruited, and the pedigrees are presented in Fig. 1A. The clinical symptoms are briefly summarized in Table 1. In general, poor dental development (Fig. 1C, E, F, I, M, P, T) and bone deformities (Fig. 1B, D, G, J, N, O, Q–S, U–X) were present in all HR patients within the recruited families. Notably, most HR patients were experiencing short stature (II-1 (family 1), I-2/II-2 (family 3), II-3 (family 5), III-9 (family 6), as well as II-1 (family 7)) and frequent fractures (family 2, 4 (Fig. 1K, L), 5, 6, 7 and 9). HR patients in family 1, 2, 3 (Fig. 1H), and 8 also displayed impaired physical mobility, and bone pain was reported in family 2, 3, and 4. In addition to these common HR characteristics, HR patients in family 5 also displayed knee valgus and scoliosis. The proband II-1 in family 9 showed kyphosis (Fig. 1Y, Z), as well as insensitivity to pain and temperature in the left arm.

**Table 1:** Clinical symptoms and information on the variants in the nine HR-affected families

Family	Patient (age)		Clinical symptoms			Variant gene		Variant type		Variant position		Nucleotide change		Amino acid change		Novelty
Family 1		I-2 (32 yr)		Loss of teeth; bone deformity; unable to walk	*PHEX*		Splicing		Exon 5		c.591A > G		(p.Gly196Alafs*16)		Reported	
		II-1 (5 yr)		Growth retardation; short stature (− 4.26 SD); Loss of teeth; bone deformity; mental retardation												
Family 2		II-1		Dental defect; bone deformity; fractures; bone pain; weakness in walking	*PHEX*		Deletion		Exon 17		c.1661_1726del		(p.Glu554_Gly575del)		Novel	
Family 3		I-2		Loss of teeth; bone deformity; short stature (− 7.52 SD)	*PHEX*		Missense; Nonsense		Exon 9; Exon 9		c.[980A > G; 1078A > T]		p.[Tyr327Cys; Lys360*]		Novel; Novel	
		II-2 (37 yr)		Loss of teeth; bone deformity; fractures; bone pain; short stature (− 8.64 SD); unable to walk												
Family 4		II-3 (34 yr)		Dental defect; bone deformity; fractures; bone pain	*PHEX*		Missense		Exon 17		c.1735G > A		(p.Gly579Arg)		Reported	
Family 5		I-2		Short stature; mild bone deformity; loss of teeth; bowing; fractures	*PHEX*		Splicing		Intron 9		c.1079 + 1G > A		(p.?)		Reported	
		II-1		Short stature; bone deformity; loss of teeth; bowing; fractures												
		II-3 (35 yr)		Short stature (− 11.10 SD); bone deformity; loss of teeth; bowing; fractures; knee valgus; scoliosis												
		II-5		Short stature; bone deformity; loss of teeth; bowing; fractures												
		III-1 (10 yr)		Bowing; knee valgus												
Family 6		II-9		Loss of teeth; bone pain	*PHEX*		Duplication		Exon 9		c.1017_1051dup		(p.Phe351Trpfs*16)		Novel	
		III-9 (29 yr)		Loss of teeth; bone deformity; fractures; short stature (− 5.67SD)												
		IV-1		Trembling in both lower limbs; foot eversion; poor balance when walking												
Family 7		II-1 (28 yr)		Loss of teeth; O-shaped legs; short stature (− 5.67SD); fractures	*PHEX*		Splicing		Intron 19		c.1965 + 1G > A		(p.?)		Reported	
Family 8		II-1 (6 yr)		X-shaped legs; dental defect; impaired physical mobility	*PHEX*		Missense		Exon 16		C.1699C > T		(p.Arg567*)		Reported	
Family 9		II-1 (34 yr)		X-shaped legs; loss of teeth; fractures; kyphosis	*SLC34A3*		Missense		Exon 12/Exon 12		c.1336G > A/c.1364 T > C		(p.Val446Ile)/(p.Leu455Pro)		Novel/Novel	

### Assessment of biochemical indicators in HR patients

Biochemical data were collected and are summarized in Table 2. It was found that the serum phosphate and serum creatinine levels were reduced in all HR patients, while β-CTX and urine phosphate levels were elevated, compared to healthy controls. Normal to high alkaline phosphatase (ALP) levels were found in patients’ serum, and very high ALP levels were detected in proband 6 (575.00 U/L), proband 8 (757.90 U/L), and proband 9 (555.00 U/L). Levels of parathyroid hormone (PTH), an important hormone that regulates calcium and phosphorus metabolism, varied between individuals, with elevated levels detected in the majority of the HR patients.

**Table 2:** Biochemical indicators of the HR probands

Characteristic	P1^e^	P3	P4	P5	P6	P8	P9	Reference values
Serum phosphate (mg/dl)	**2.05** (32 yr)	**1.80** (37 yr)	–	**2.20** (41 yr)	**2.57** (29 yr)	**1.98** (3 yr)	**1.55** (31 yr)	4.81–8.22 (< 1 yr) 3.88–6.51 (1–3 yr) 3.72–5.58 (4–11 yr) 2.95–5.43 (12–15 yr) 2.79–4.65 (> 15 yr)
Serum calcium (mg/dl)	9.52	8.92	–	8.72	9.04	9.60	8.80	8.52–10.8
Serum alkaline phosphatase (U/L)	104.00	**166.00**	63.60	118.00	**575.00**	**757.90**	**555.00**	45.00–125.00
Serum creatinine (mg/dl)	**0.38**	**0.31**	–	–	–	**0.22**	**0.60**	0.67–1.18
β-CTX (ng/ml)^a^	–	–	–	**0.78**	–	–	**1.96**	0.26–0.51
T-25OHD (ng/ml)^b^	13.60	–	–	13.70	–	29.31	18.00	8.00–50.00
VitD3 (µg/L)^c^	–	–	–	45.65	–	26.30	**173.82**	19.00–57.60
Urine phosphate (mg/dl)	**35.65**	–	–	**17.98**	–	**30.72**	**42.29**	3.41–5.58
Urine calcium (mg/dl)	8.56	–	–	6.72	0.32	–	54.20	–
Urine creatinine (mg/dl)	–	–	–	–	77.18	74.47	–	28.81–226
PTH (pg/ml)^d^	**84.60**	–	–	61.20	**136.40**	**126.70**	**14.50**	15.00–65.00

Numbers in bold represent abnormal values

^a^β-CTX = β-C-terminal telopeptides

^b^T-25OHD = Serum total 25-hydroxyvitamin D

^c^VitD3 = Serum 1,25(OH)_2_ vitamin D3

^d^PTH = Parathyroid hormone

^e^Pn (n = 1, 3, 4, 5, 6, 8, 9) represents the proband number; data of probands 2 and 7 were not available

### Identification of variants in *PHEX* and *SLC34A3*

**Fig. 2:**
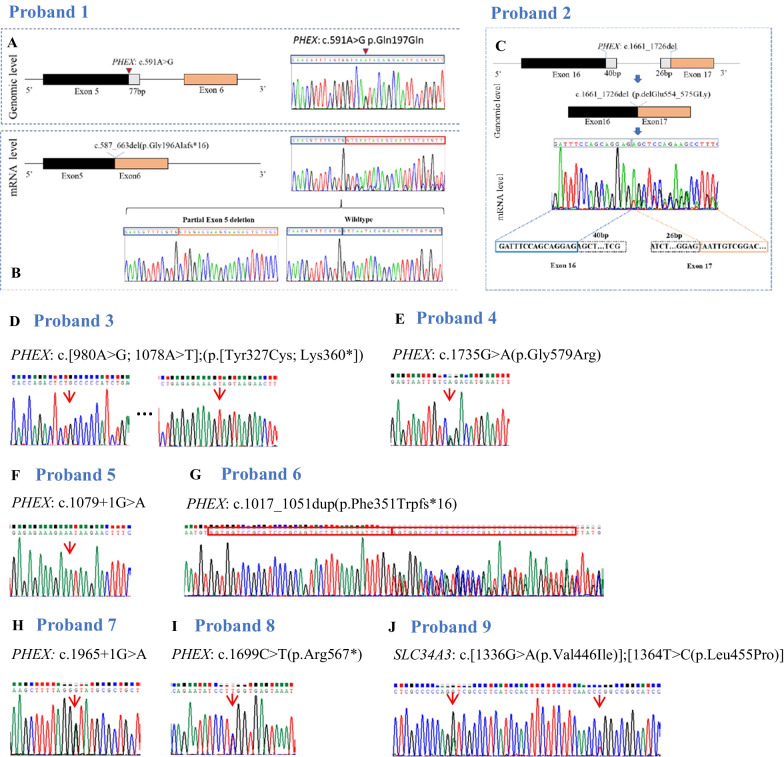
Variant analysis of Families 1–9. **A** In proband 1, a synonymous variant c.591A > G(p.Gln197Gln) was identified in genomic DNA. **B** Sanger sequencing at the mRNA level indicated that part of exon 5 was deleted, caused by variant c.591A > G, leading to a frameshift inducing a premature stop codon (p.Gly196Alafs*16). **C** In proband 2, Sanger sequencing confirmed that c.1661_1726del did not induce alternative splicing. **D–J** Sanger sequencing results of probands 3**–**9

Sanger sequencing of the exons and associated splicing sites, as well as both UTR regions of the *PHEX* gene revealed nine *PHEX* variants in family 1–8 (Table 1, Fig. 2). Among these nine *PHEX* variants, four were novel (c.1661_1726del, c.980A > G, c.1078A > T, and c.1017_1051dup), and five were previously reported in HGMD (c.591A > G, c.1735G > A, c.1079 + 1G > A, c.1965 + 1G > A, and c.1699C > T). All families carrying *PHEX* variants exhibited an X-linked dominant inheritance, with the exceptions of family 2, 4, 7, and 8, in which de novo variants were detected. In family 1, although variant c.591A > G (p.Gln197Gln) was supposed to be a synonymous variant, sequencing at the mRNA level identified the deletion of 77 bp at the 3′ region of exon 5 (Fig. 2A, B). mRNA sequencing was also conducted in family 2 (Fig. 2C). This confirmed that the variant c.1661_1726del did not lead to alternative splicing, but rather to the deletion of 66 bp spanning from 3′ of exon 16 to 5′ of exon 17 (Fig. 2C). A cis double variant c.[980A > G; 1078A > T] was found in family 3 (Fig. 2D), with both variants being inherited from the mother. In family 6, the proband (III-9) with HR (c.1017_1051dup in *PHEX*) married III-8 with hereditary spastic paraplegia harboring the variant c.715C > T(p.Arg239Cys) in *ALT1*. Their daughter (IV-1) inherited both variants and exhibited the phenotypes of both diseases.

The pathogenic gene of family 9 was *SLC34A3*, and proband 9 was a heterozygote of variants c.1336G > A(p.Val446Ile) and c.1364T > C(p.Leu455Pro) (Table 1). The variant c.1336G > A was inherited from the father, while c.1364 T > C was inherited from the mother. The younger brother of the proband (II-2) inherited neither of these variants from the parents (Fig. 2J, Additional file 1: Fig. S2).

### Bioinformatic analysis of the variants

**Fig. 3:**
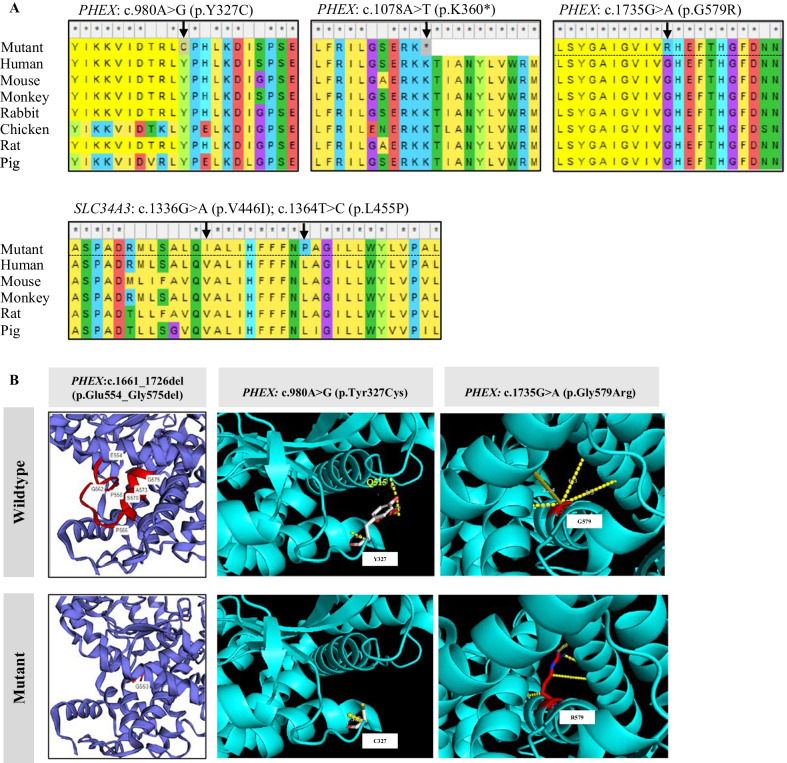
Bioinformatic analysis of the variants. **A** Evolutionary conservation of amino acids in the protein products of PHEX and SLC34A3. Variant sites are indicated by black arrows. **B** Comparison of the 3D structures of mutant proteins with the wild type. The 3D structures of the protein products of c.1661_1726del(p.Glu554_Gly575del) (left), missense variant c.980A > G(p.Tyr327Cys) (middle), and missense variant c.1735G > A(p.Gly579Arg) (right) are compared with the corresponding wild type

It was confirmed that all point mutation sites, p.Y327C, p.K360*, and p.G579R in *PHEX*, and p.V446I and p.L455P in *SLC34A3*, were highly evolutionarily conserved (Fig. 3A). To predict the likelihood of pathogenicity, two online tools (PROVEAN and PolyPhen-2) were used and the predicted scores are summarized in Additional file 1: Table S3. In addition, the splice site variants were predicted as “most probably affecting splicing” by Human Splicing Finder (version 3.1) (Additional file 1: Table S3). The 3D structure of the PHEX protein was modeled by Phyre2 for the deletion c.1661_1726del in *PHEX*. In comparison to the wild-type PHEX protein, the mutant PHEX protein lacked an α-helix (5′ of exon 17) and a β-sheet (3′ of exon 16) (Fig. 3B), which altered its 3D conformation. The 3D structure of the PHEX protein was also modeled by Swiss-Model and the functional consequences of the missense variants c.980A > G(p.Tyr327Cys) and c.1735G > A(p.Gly579Arg) were simulated by Pymol (Fig. 3B). This analysis indicated altered interactions of the substituted amino acids with their neighboring residues.

## Discussion

**Fig. 4:**
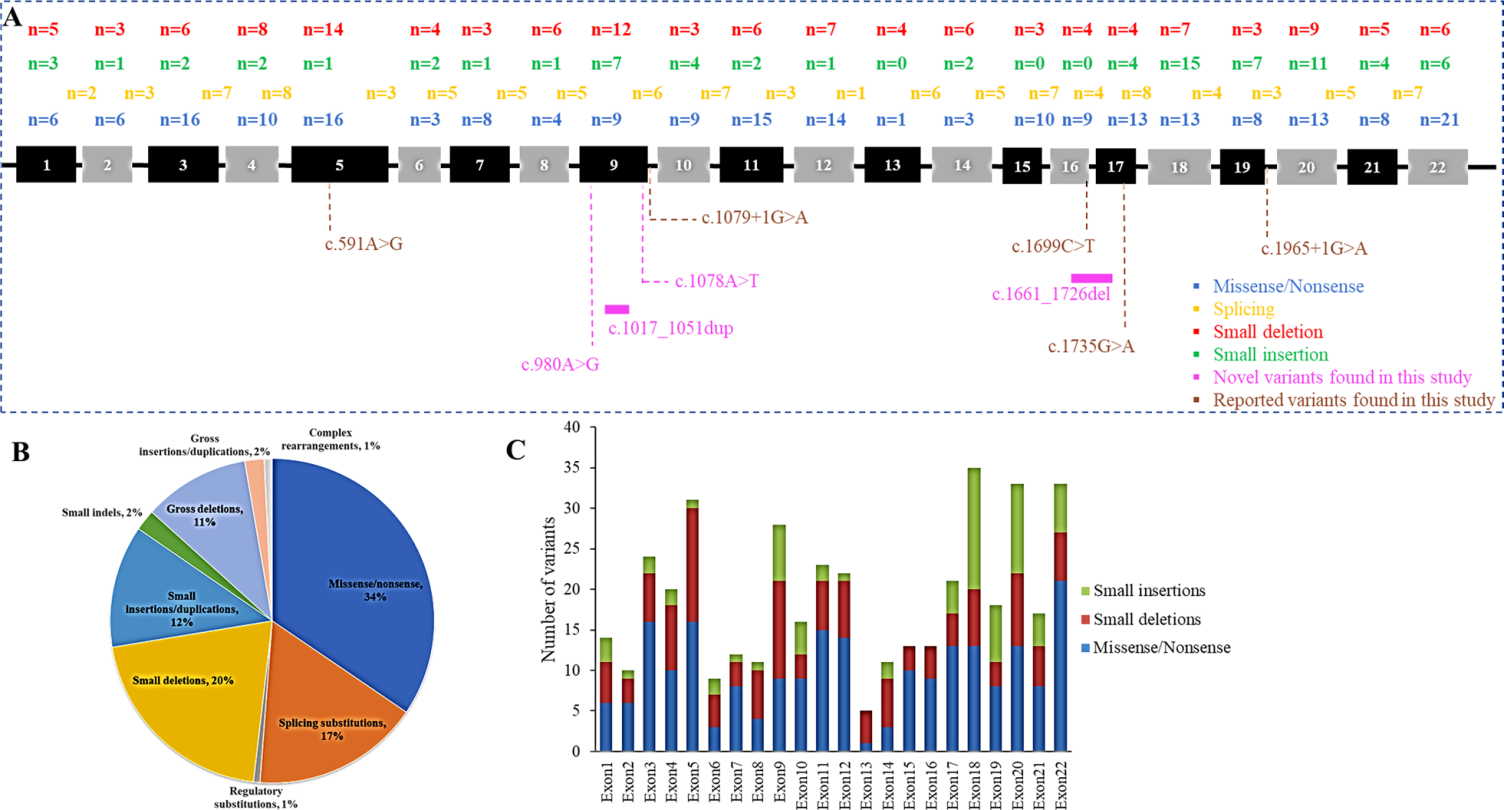
Genotypic characterization of *PHEX* mutations. **A** Mutation spectrum of *PHEX*. Each numbered box represents a corresponding exon in *PHEX*. Numbers of missense/nonsense mutations (blue), splicing substitutions (yellow), small deletions (red), and small insertions (green) are presented above the 22 exons. The novel variants found in this study are listed below in pink, while previously reported variants found in this study are listed below in brown. **B** Distribution of mutations in *PHEX* (HGMD Professional, release 2020.04). **C** Distribution of mutations in each exon of *PHEX* (HGMD Professional, release 2020.04)

To date, 623 variants have been identified in *PHEX*, including 215 missense/nonsense mutations (34%), 104 splicing substitutions (17%), 4 regulatory substitutions (1%), 128 small deletions (20%), 76 small insertions/duplications (12%), 13 small indels (2%), 66 gross deletions (11%), 12 gross insertions/duplications (2%), and 5 complex rearrangements (1%) (Fig. 4B). Point mutations were predominant, constituting 51% of all mutations, followed by small copy number variations (34%). Here, the pathogenic mutations in each exon across the *PHEX* gene identified to date have been summarized (Fig. 4A, C), and it was confirmed that the mutation spectrum spanned the whole gene without any hotspot regions [17, 27, 28].

Common consequences of gene mutations are dysfunction of protein, frameshift, truncated protein, protein redundancy, and lack of protein product resulting from mRNA degradation. It has been reported that most cases of XLHR induced by *PHEX* mutations are caused by the generation of a truncated protein or by the translation of a dysfunctional PHEX product [29], and this was further confirmed in the current study. (i) It has previously been reported that about 85% of *PHEX* mutations result in a truncated PHEX protein [22]. Three novel variants identified in this study, c.1661_1726del(p.Glu554_Gly575del), c.1078A > T(p.Lys360*), and c.1017_1051dup(p.Phe351Trpfs*16), as well as two previously reported variants c.591A > G(p.Gly196Alafs*16) and c.1699C > T(p.Arg567*), resulted in a truncated PHEX protein and the characteristic HR phenotype. The synonymous variant c.591A > G(p.Gln197Gln) resulted in a frameshift-induced premature termination codon (p.Gly196Alafs*16) (Fig. 2A, B), which was caused by the recognition of an alternative splice donor site “GT” located 3 bp upstream of the mutant site in exon 5. This is in line with findings reported by Liao et al. [30]. (ii) Some *PHEX* mutations, such as missense mutations and a few splicing mutations, resulted in dysfunction of the PHEX protein. Most of the missense mutations found in *PHEX*, although located at different sites, affect post-translational modification of the protein, thus leading to loss of protein function [27]. For example, the reported missense mutation c.1735G > A(p.Gly579Arg) identified in family 4 [31, 32] was located at the extracellular C-terminal region, which could affect the secondary structure of the PHEX protein by disrupting disulfide bonds [31]. The predicted 3D structure of the PHEX protein (Fig. 3B) also indicated that Gly579Arg, a change involving substitution of the short-side-chain glycine for the long-side-chain arginase, would possibly push away the interacting α-helix, having a negative effect on protein function. Compared with the severely impacted physical mobility reported in a Turkish family with the same variant [31], the clinical symptoms of the HR patients in this study were limited to mild bone deformities.

The proband in family 3 carried a missense variant c.980A > G(p.Tyr327Cys) and a nonsense variant c.1078A > T(p.Lys360*) (Fig. 2D), both located on the same allele and inherited from his mother (Additional file 1: Fig. S1C). It has previously been shown that a truncated protein generated from a nonsense mutation can cause abnormal bone mineralization and hypophosphatemia [33]. The above-mentioned nonsense variant was therefore predicted to be pathogenic, which was supported by in silico tools (Additional file 1: Table S3). Meanwhile, for the novel missense variant c.980A > G also located in exon 9, there were conflicting results on its pathogenicity (Additional file 1: Table S3). According to Gaucher et al. [17], missense mutations at highly conserved sites encoding residues in the interior of the PHEX protein could alter protein folding and trafficking. The missense variant c.980A > G(p.Tyr327Cys) resulted in an exchanged of tyrosine with cysteine, suggesting a potential role in disulfide bond formation and protein folding [31, 34]. In line with this, the predicted 3D protein structure and residue interactions (Fig. 3B) suggested that the wild-type tyrosine closely interacted with glutamine 515 at the neighboring α-helix via a hydrogen bond. However, the mutant cysteine could not form this hydrogen bond and did not interact with the α-helix, potentially changing the stability of the protein. The above findings show that the nonsense variant in family 3 contribute to the development of HR, and indicate a potential role also for the missense variant.

No genotypic and phenotypic correlation was found in this study, which is in line with previous findings [22, 35]. Nevertheless, the reported variants were concentrated in exons 18–22 (Fig. 4C), indicating that this extracellular C-terminal region is essential for PHEX protein function [36]. It has been reported that patients with variants at the C-terminal region or truncated proteins exhibited more severe HR phenotypes [35], and variants leading to truncated PHEX proteins generally corresponded to decreased tubular reabsorption of phosphate and 1,25(OH)_2_D [37]. Out of the four novel *PHEX* variants found in the present study, the first two (located in exon 9) resulted in a truncated PHEX product, the third (located in exon 9) resulted in amino acid alteration, and the fourth (located in exon 17) resulted in a truncated protein. Zhang et al. reported that the missense variants were clustered in exons 15, 17, 19, and 20 in *PHEX* [22], while the missense variant identified in this study was located in exon 9. This identifies exon 9 as an important novel player in the progression of HR.

The HR patients included in this study had a significantly shorter stature (ranging from − 4.26 to − 11.10 SD) than previously reported HR patients in Chinese cohort (− 2.70 ± 1.60 SD) [22]. Possible reasons for this are chronical nutritional deficiencies and extended periods of untreated disease progression due to patients’ living in remote rural areas with limited access to medical facilities. Noteworthy is that HR patients with a more severe phenotype often had a family history. As the majority of the HR patients recruited in this study also had suffered frequent fractures (Table 1), which can be misdiagnosed as osteogenesis imperfecta, clinical examinations combined with molecular testing are crucial for a precise diagnosis.

In this study, one compound heterozygous variant was found in *SLC34A3* in family 9 (Fig. 2J, Table 1). *SLC34A3* is expressed in tubule cells and is responsible for the maintenance of phosphate homeostasis [38]. In previous studies, phenotypic heterogeneity was found in patients with *SLC34A3* variants: approximately 25% of HHRH patients did not exhibit rickets and half lacked renal calcification [39]. It was also shown that heterozygous carriers presented milder skeletal phenotypes than homozygous ones [40]. Typical HR symptoms were noted in the patient with *SLC34A3* variants in this study, while the heterozygous carriers (I-1 and I-2) barely showed any noticeable phenotypes. A study with a large sample size is needed to obtain a comprehensive understanding of the genotype/phenotype correlation in HHRH.

## Conclusion

In summary, we identified nine pathogenic variants in *PHEX* and one compound heterozygous variant in *SLC34A3* in nine Chinese families with HR. These variants included four novel variants in *PHEX* and two novel variants in *SLC34A3*. The current study confirms the various variant types associated with HR and provides new insight that expands our understanding of the function of *PHEX*.

## Supplementary Information

**Additional file 1.** Supplementary materials.

## Data Availability

The data that support the findings of this study have been deposited into CNGB Sequence Archive (CNSA) [41] of China National GeneBank Database (CNGBdb) [42] with accession number CNP0001901.
