# Supplementary material for: Identification of six novel variants from nine Chinese families with hypophosphatemic rickets
Source: BMC Med Genomics. 2022 Jul 16;15:161. doi: 10.1186/s12920-022-01305-w (PMC9287957; doi:10.1186/s12920-022-01305-w)
Supplement: Supplementary file 1 — Additional file 1. Supplementary materials. [file 12920_2022_1305_MOESM1_ESM.docx]

**Table S1. Primers designed for PCR and RT-PCR**

| **Primer name** | **Sequence (5' → 3')** | **Size （bp)** | **Tm (℃)** |
| --- | --- | --- | --- |
| *PHEX*-Exon 1-Fw | TGAGAGAAGAGCCTGCCAAAC | 545 | 58 |
| *PHEX*-Exon 1-Rv | GATACACCACCTATGAACGCAG |  |  |
| *PHEX*-Exon 2-Fw | CGTGACATTGAACCAACTGGG | 447 | 58 |
| *PHEX*-Exon 2-Rv | TAACAACGCTTAGAATACCACAG |  |  |
| *PHEX*-Exon 3-Fw | GGCTTGGAAACTGGTTGATA | 545 | 58 |
| *PHEX*-Exon 3-Rv | TTCCTTTGCTTCCAATCTCA |  |  |
| *PHEX*-Exon 4-Fw | CCTCATGCAACTTGGAATCT | 537 | 58 |
| *PHEX*-Exon 4-Rv | GCCCTGCTGACTTTGTCTAA |  |  |
| *PHEX*-Exon 5-Fw | CTAGTGTGCTGATCCAGTTTGC | 370 | 58 |
| *PHEX*-Exon 5-Rv | GCAGCATGAGTCTCTTTCCC |  |  |
| *PHEX*-Exon 6-Fw | AGGGGCAGGTCTCTGATAGT | 469 | 58 |
| *PHEX*-Exon 6-Rv | TTGTACAGCCTTGATGAGCA |  |  |
| *PHEX*-Exon 7-Fw | CTTGATGGCTGGACATCTCT | 581 | 58 |
| *PHEX*-Exon 7-Rv | GTGGAAGGAATGAGGAGCTT |  |  |
| *PHEX*-Exon 8-Fw | TTTTTCTCTTCCCCGAGTTG | 502 | 58 |
| *PHEX*-Exon 8-Rv | GAGCCAATGCCAACAATTAC |  |  |
| *PHEX*-Exon 9-Fw | GATGCTCAATCTACTCTATCTGCC | 379 | 58 |
| *PHEX*-Exon 9-Rv | CAGTTCATCACAAAGGACACCG |  |  |
| *PHEX*-Exon 10-Fw | TGTATGAGTAAGAGGTCCCTCGATG | 321 | 58 |
| *PHEX*-Exon 10-Rv | CTCCCCCTGTCTAATCCCTAAAGAT |  |  |
| *PHEX*-Exon 11-Fw | TTCAGGTTGTTTGAATTGTTTTCAG | 255 | 58 |
| *PHEX*-Exon 11-Rv | GATCTGGCTAAATTGCCATTATTTT |  |  |
| *PHEX*-Exon 12-Fw | AGCATGGAGTCAAGCTGAAAGA | 306 | 58 |
| *PHEX*-Exon 12-Rv | TGTCAAGCATGAACATCCATTAAA |  |  |
| *PHEX*-Exon 13-Fw | AGATGAAGGGCGCATTTCTACA | 261 | 58 |
| *PHEX*-Exon 13-Rv | TCACCAGTTTTAATTGCTAGGAC |  |  |
| *PHEX*-Exon 14-Fw | GAACAATGATGTTGTGGTTTGTTT | 192 | 58 |
| *PHEX*-Exon 14-Rv | AGACTCCGCTTCTCACCAATG |  |  |
| *PHEX*-Exon 15-Fw | AGCCATGCTGTGTTTGTCTTTG | 218 | 58 |
| *PHEX*-Exon 15-Rv | CTTACCCTCCATCATAGTCATG |  |  |
| *PHEX*-Exon 16-Fw | CCAGGTACTCATCATTGAATC | 226 | 58 |
| *PHEX*-Exon 16-Rv | CCATGGCTTCTTTCTGCTGA |  |  |
| *PHEX*-Exon 17-Fw | GCAGTTTATCTTGGCTTTCC | 320 | 58 |
| *PHEX*-Exon 17-Rv | GCAAGACACGGTGAAAAGTT |  |  |
| *PHEX*-Exon 18-Fw | CTCATTTTTAAGGTGCTCTTTGTTC | 436 | 58 |
| *PHEX*-Exon 18-Rv | GCAAGCTAATGTCCAAATGAGG |  |  |
| *PHEX*-Exon 19-Fw | CCTCTTGCTGAATGATAGTTGACC | 288 | 58 |
| *PHEX*-Exon 19-Rv | TCAATGGGGAGACACACTTCTAT |  |  |
| *PHEX*-Exon 20-Fw | TGAGCAAAGAGAAAAACCCACCGTT | 219 | 58 |
| *PHEX*-Exon 20-Rv | GGAGCAAACTCAAGTCCTGCATCTC |  |  |
| *PHEX*-Exon 21-Fw | TCCTCAGTATAATTTGGAGCAG | 249 | 58 |
| *PHEX*-Exon 21-Rv | CTGGTAGAGCCCTTGGATGG |  |  |
| *PHEX*-Exon 22-Fw | CAACCTTCTTTCTAGCAATATTCTG | 700 | 58 |
| *PHEX*-Exon 22-Rv | GAGGAATGCTGTGTAATTACTG |  |  |
| **RT-PCR** |  |  |  |
| *PHEX-*E3-7-RT-Fw | TCTGTCTGTGGATCCTTGTG | 602 | 58 |
| *PHEX-*E3-7-RT-Rv | TCATGTCATGCTCTGCTCTG |  |  |
| *PHEX-*E12-22-RT-Fw | AAAGTGCCCTCCCTTATGTTG | 1103 | 58 |
| *PHEX-*E12-22-RT-Rv | CTAAAGCAATGGGCGATGAA |  |  |

**Table S2. 184 genes included in the customized sequencing panel**

| **Gene** | **Reference Sequence (GRCh38)** | **Length** | **Phenotype** |
| --- | --- | --- | --- |
| *COL1A1* | NC_000017.11 (50184096..50201649, complement) | 17,544 | Osteogenesis imperfecta |
| *COL1A2* | NC_000007.14 (94394561..94431232) | 36,672 | Osteogenesis imperfecta |
| *IFITM5* | NC_000011.10 (298200..299526, complement) | 1,326 | Osteogenesis imperfecta |
| *SERPINF1* | NC_000017.11 (1761925..1777574) | 15,650 | Osteogenesis imperfecta |
| *CRTAP* | NC_000003.12 (33113958..33147773) | 33,795 | Osteogenesis imperfecta |
| *P3H1* | NC_000001.11 (42746335..42767084, complement) | 20,750 | Osteogenesis imperfecta |
| *PPIB* | NC_000015.10 (64155815..64163155, complement) | 7,394 | Osteogenesis imperfecta |
| *SERPINH1* | NC_000011.10 (75562056..75572804) | 10,749 | Osteogenesis imperfecta |
| *FKBP10* | NC_000017.11 (41812710..41823217) | 10,508 | Osteogenesis imperfecta |
| *PLOD2* | NC_000003.12 (146069437..146161495, complement) | 91,728 | Osteogenesis imperfecta |
| *BMP1* | NC_000008.11 (22165140..22212326) | 47,591 | Osteogenesis imperfecta |
| *SP7* | NC_000012.12 (53326575..53344793, complement) | 9,808 | Osteogenesis imperfecta |
| *TMEM38B* | NC_000009.12 (105694525..105776611) | 82,069 | Osteogenesis imperfecta |
| *WNT1* | NC_000012.12 (48978453..48982613) | 4,161 | Osteogenesis imperfecta |
| *CREB3L1* | NC_000011.10 (46277638..46321422) | 43,785 | Osteogenesis imperfecta |
| *SPARC* | NC_000005.10 (151661096..151687054, complement) | 26,070 | Osteogenesis imperfecta |
| *MBTPS2* | NC_000023.11 (21839538..21885423) | 45,886 | Osteogenesis imperfecta |
| *SEC24D* | NC_000004.12 (118722823..118838683, complement) | 113,316 | Osteogenesis imperfecta |
| *LRP5* | NC_000011.10 (68298866..68449275) | 136,667 | Osteogenesis imperfecta |
| *P4HB* | NC_000017.11 (81843158..81860668, complement) | 17,536 | Osteogenesis imperfecta |
| *PLS3* | NC_000023.11 (115560850..115650861 | 90,012 | Osteogenesis imperfecta |
| *NTRK1* | NC_000001.11 (156815750..156881850) | 66,101 | Congenital insensitivitv to pain with anhidrosis |
| *NGF* | NC_000001.11 (115285915..115338253, complement) | 52,339 | Congenital insensitivitv to pain with anhidrosis |
| *NDN* | NC_000015.10 (23685407..23687303, complement) | 1,897 | Congenital insensitivitv to pain with anhidrosis |
| *SCN9A* | NC_000002.12 (166195185..166375987, complement) | 180,803 | Congenital insensitivitv to pain with anhidrosis |
| *SCN11A* | NC_000003.12 (38845764..39051945, complement) | 206,223 | Congenital absence of pain |
| *DMD* | NC_000023.11 (31119219..33339609, complement) | 2,220,391 | Pseudo hypertrophic muscular dystrophy |
| *EDA* | NC_000023.11 (69616067..70039472) | 423,406 | Anhidrotic ectodermal dysplasia |
| *EDAR* | NC_000002.12 (108894471..108989372, complement) | 94,902 | Hypohidrosis ectodermal dysplasia |
| *RETREG1* | NC_000005.10 (16473038..16617096, complement) | 144,021 | Indifference to pain |
| *GJB6* | NC_000013.11 (20221962..20232395, complement) | 10,434 | Ectodermal dysplasia |
| *EDARADD* | NC_000001.11 (236394380..236484708) | 90,329 | Ectodermal dysplasia |
| *KRT85* | NC_000012.12 (52360006..52367525, complement) | 7,520 | Ectodermal dysplasia |
| *MSX1* | NC_000004.12 (4859665..4863936) | 4,272 | Ectodermal dysplasia |
| *GRHL2* | NC_000008.11 (101492439..101678104) | 185,666 | Ectodermal dysplasia |
| *IKBKG* | NC_000023.11 (154542240..154565046) | 22,807 | Incontinentia pigmenti |
| *DKC1* | NC_000023.11 (154762742..154777689) | 14,948 | X-linked dyskeratosis congenita |
| *LPP* | NC_000003.12 (188152152..188890671) | 738,520 | Lipoma |
| *OAT* | NC_000010.11 (124397303..124418976, complement) | 21,674 | Gyrate atrophy of choroid and retina with or without ornithinemia |
| *FZD4* | NC_000011.10 (86945675..86955398, complement) | 9,724 | Exudative vitreoretinopathy 1 |
| *OSMR* | NC_000005.10 (38845858..38945579) | 89,643 | Primary localized cutaneous amyloidosis |
| *IL31RA* | NC_000005.10 (55840334..55922854) | 71,476 | Primary localized cutaneous amyloidosis |
| *KIT* | NC_000004.12 (54657928..54740715) | 82,798 | Mottled disease |
| *SNAI2* | NC_000008.11 (48917677..48921440, complement) | 3,973 | Mottled disease |
| *ABCC6* | NC_000016.10 (16149565..16223617, complement) | 2,285 | Pseudoxanthoma elasticum |
| *XYLT1* | NC_000016.10 (17102324..17470881, complement) | 368,558 | Pseudoxanthoma elasticum |
| *XYLT2* | NC_000017.11 (50346032..50361185) | 15,154 | Pseudoxanthoma elasticum |
| *GGCX* | NC_000002.12 (85544720..85561534, complement) | 16,815 | Pseudoxanthoma elasticum |
| *TSC1* | NC_000009.12 (132891348..132945269, complement) | 53,286 | Tuberous sclerosis |
| *TSC2* | NC_000016.10 (2047804..2088720) | 40,826 | Tuberous sclerosis |
| *STS* | NC_000023.11 (7147252..7354643) | 207,392 | X-linked ichthyosis |
| *KRT1* | NC_000012.12 (52674736..52680407, complement) | 5,672 | Epidermolytic palmoplantar keratoderma |
| *KRT9* | NC_000017.11 (41565841..41572058, complement) | 6,217 | Epidermolytic palmoplantar keratoderma |
| *FERMT1* | NC_000020.11 (6074845..6123609, complement) | 48,700 | Kindler syndrome |
| *NF1* | NC_000017.11 (31094927..31377677) | 282,751 | Neurofibromatosis type 1 |
| *NF2* | NC_000022.11 (29603556..29698600) | 95,045 | Neurofibromatosis type 2 |
| *TYR* | NC_000011.10 (89177565..89295759) | 118,195 | Albinism |
| *OCA2* | NC_000015.10 (27719008..28099342, complement) | 380,335 | Albinism |
| *MC1R* | NC_000016.10 (89917879..89920977) | 3,099 | Albinism |
| *GPR143* | NC_000023.11 (9725413..9786260, complement) | 40,553 | Albinism |
| *TYRP1* | NC_000009.12 (12693375..12710266) | 16,882 | Albinism |
| *MITF* | NC_000003.12 (69739435..69968337) | 228,903 | Albinism |
| *SLC45A2* | NC_000005.10 (33944616..33984675, complement) | 40,060 | Albinism |
| *SLC24A5* | NC_000015.10 (48120972..48142392) | 21,421 | Albinism |
| *LRMDA* | NC_000010.11 (75431646..76557375) | 1,125,730 | Albinism |
| *FGFR3* | NC_000004.12 (1793299..1808872) | 15,561 | Achondroplasia |
| *COMP* | NC_000019.10 (18782773..18791305, complement) | 8,542 | Pseudochondrodysplasia |
| *TRIP11* | NC_000014.9 (91965991..92040134, complement) | 74,150 | Chondrogenesis IA type |
| *SLC26A2* | NC_000005.10 (149960737..149987400) | 26,672 | Chondrogenesis IB type |
| *COL2A1* | NC_000012.12 (47972965..48006212, complement) | 33,248 | Chondrogenesis II type |
| *EVC2* | NC_000004.12 (5562408..5709548, complement) | 177,628 | Ectodermal dysplasia |
| *EVC* | NC_000004.12 (5711197..5829043) | 117,847 | Ectodermal dysplasia |
| *SHOX* | NC_000023.11 (624344..659411) | 35,068 | Chondrogenesis |
| *COL9A2* | NC_000001.11 (40300487..40317653, complement) | 17,167 | Multiple epiphyseal dysplasia type 2 |
| *COL9A3* | NC_000020.11 (62817062..62841159) | 24,098 | Multiple epiphyseal dysplasia type 3 |
| *MATN3* | NC_000002.12 (19992052..20012694, complement) | 20,643 | Multiple epiphyseal dysplasia type 5 |
| *COL9A1* | NC_000006.12 (70216040..70303083, complement) | 87,044 | Multiple epiphyseal dysplasia type 6 |
| *SLCO2A1* | NC_000003.12 (133932696..134030076, complement) | 97,381 | Pachydermoperiostosis |
| *HPGD* | NC_000004.12 (174490177..174522898, complement) | 32,722 | Pachydermoperiostosis |
| *CTSK* | NC_000001.11 (150796208..150808441, complement) | 12,234 | Developmental disorder of compact bone |
| *COL10A1* | NC_000006.12 (116118909..116219937, complement) | 99,790 | Schmid type epiphyseal cartilage dysplasia |
| *RMRP* | NC_000009.12 (35657751..35658018, complement) | 268 | McKusick type epiphyseal cartilage dysplasia |
| *PTHR1* | NC_000003.12 (46877689..46903799) | 26,110 | Jansen type epiphyseal cartilage dysplasia |
| *SBDS* | NC_000007.14 (66987677..66995696, complement) | 7,899 | Dysplasia of the pancreas with abnormal epiphyseal cartilage |
| *TPM2* | NC_000009.12 (35681993..35690056, complement) | 7,900 | Distal arthrogryposis |
| *TNNT3* | NC_000011.10 (1919551..1938706) | 19,145 | Distal arthrogryposis |
| *TNNI2* | NC_000011.10 (1839003..1841680) | 2,690 | Distal arthrogryposis |
| *MYH3* | NC_000017.11 (10628526..10678347, complement) | 28,783 | Distal arthrogryposis |
| *MYH8* | NC_000017.11 (10390325..10421950, complement) | 31,629 | Distal arthrogryposis |
| *PIEZO2* | NC_000018.10 (10670237..11149585, complement) | 477,062 | Distal arthrogryposis |
| *ECEL1* | NC_000002.12 (232479827..232487859, complement) | 8,033 | Distal arthrogryposis |
| *SYNE1* | NC_000006.12 (152121684..152637399, complement) | 1,019 | Distal arthrogryposis |
| *IHH* | NC_000002.12 (219054420..219060516, complement) | 6,358 | Brachydactyly syndrome |
| *BMPR1B* | NC_000004.12 (94757977..95158453) | 400,477 | Brachydactyly syndrome |
| *BMP2* | NC_000020.11 (6768098..6780280) | 12,581 | Brachydactyly syndrome |
| *GDF5* | NC_000020.11 (35433347..35454746, complement) | 21,400 | Brachydactyly syndrome |
| *HOXD13* | NC_000002.12 (176087487..176095938) | 8,452 | Brachydactyly syndrome |
| *ROR2* | NC_000009.12 (91722596..91950206, complement) | 227,611 | Brachydactyly syndrome |
| *NOG* | NC_000017.11 (56593699..56595590) | 1,892 | Brachydactyly syndrome |
| *GDF5* | NC_000020.11 (35433347..35454746, complement) | 21,400 | Brachydactyly syndrome |
| *PTHLH* | NC_000012.12 (27958084..27972864, complement) | 16,104 | Brachydactyly syndrome |
| *PDE3A* | NC_000012.12 (20369245..20688579) | 314,863 | Brachydactyly syndrome |
| *CHSY1* | NC_000015.10 (101175723..101251932, complement) | 76,210 | Brachydactyly syndrome |
| *PITX1* | NC_000005.10 (55825044..55836193, complement) | 6,541 | Brachydactyly syndrome |
| *HDAC6* | NC_000023.11 (48801377..48824982) | 23,606 | Brachydactyly syndrome |
| *TBC1D24* | NC_000016.10 (2475104..2509669) | 34,566 | Brachydactyly syndrome |
| *TRPV4* | NC_000012.12 (109783087..109833407, complement) | 50,321 | Brachydactyly syndrome |
| *RUNX2* | NC_000006.12 (45328317..45551082) | 335,891 | Brachydactyly syndrome |
| *SHH* | NC_000071.6 (155799984..155812273, complement) | 12,288 | Polydactyly syndrome |
| *GLI3* | NC_000079.6 (41960949..42237019, complement) | 276,064 | Polydactyly syndrome |
| *FBLN1* | NC_000022.11 (45502839..45601134) | 98,296 | Polydactyly syndrome |
| *SALL1* | NC_000016.10 (51135975..51151272, complement) | 16,412 | Polydactyly syndrome |
| *FGFR2* | NC_000010.11 (121478330..121598458, complement) | 120,327 | Polydactyly syndrome |
| *FGFR3* | NC_000004.12 (1793299..1808872) | 15,561 | Polydactyly syndrome |
| *FGF10* | NC_000005.10 (44301655..44389706, complement) | 88,126 | Polydactyly syndrome |
| *LRP4* | NC_000011.10 (46854715..46918622, complement) | 63,908 | Polydactyly syndrome |
| *GREM1* | NC_000015.10 (32718004..32734669) | 16,730 | Polydactyly syndrome |
| *FMN1* | NC_000015.10 (32765544..33194765, complement) | 429,222 | Polydactyly syndrome |
| *FAM58A* | NC_000023.11 (153587925..153599177, complement) | 11,253 | Polydactyly syndrome |
| *NEK1* | NC_000004.12 (169393270..169612629, complement) | 219,360 | Polydactyly syndrome |
| *WDR35* | NC_000002.12 (19910260..19990123, complement) | 79,864 | Polydactyly syndrome |
| *DYNC2H1* | NC_000011.10 (103109431..103479863) | 370,433 | Polydactyly syndrome |
| *PIK3R2* | NC_000019.10 (18153178..18170533) | 17,356 | Polydactyly syndrome |
| *AKT3* | NC_000001.11 (243488233..243851079, complement) | 362,847 | Polydactyly syndrome |
| *TRPS1* | NC_000008.11 (115408496..115669637, complement) | 261,138 | Polydactyly syndrome |
| *EXT1* | NC_000008.11 (117799363..118111819, complement) | 314,324 | Polydactyly syndrome |
| *WDR60* | NC_000007.14 (158839245..158958695) | 119,446 | Polydactyly syndrome |
| *LMBR1* | NC_000007.14 (156669012..156893208, complement) | 219,855 | Polydactyly syndrome |
| *GJA1* | NC_000006.12 (121435577..121449744) | 14,168 | Syndactyly syndrome |
| *FGFR1* | NC_000008.11 (38411138..38468834, complement) | 57,697 | Missing finger syndrome |
| *WNT10B* | NC_000012.12 (48965340..48979587, complement) | 6,519 | Missing finger syndrome |
| *TP63* | NC_000003.12 (189596746..189897279) | 300,165 | Cleft hand/foot deformity |
| [*DLX5*](http://www.genenames.org/cgi-bin/gene_symbol_report?match=DLX5) | NC_000007.14 (97020390..97024831, complement) | 4,442 | Cleft hand/foot deformity |
| *DSS1* | NC_000007.14 (96481626..96709891, complement) | 21,125 | Cleft hand/foot deformity |
| [*WNT10B*](http://www.genenames.org/cgi-bin/gene_symbol_report?match=WNT10B) | NC_000012.12 (48965340..48979587, complement) | 6,519 | Cleft hand/foot deformity |
| [*FBXW4*](http://www.genenames.org/cgi-bin/gene_symbol_report?match=FBXW4) | NC_000010.11 (101610664..101695295, complement) | 84,632 | Cleft hand/foot deformity |
| *DLX6* | NC_000007.14 (97005978..97011040) | 5,063 | Cleft hand/foot deformity |
| *CLCN5* | NC_000023.11(49922615..50099235) | 176,621 | Hypophosphatemic rickets |
| *DMP1* | NC_000004.12(87650302..87664361) | 14,060 | Hypophosphatemic rickets, AR |
| *CYP27B1* | NC_000012.12(57762334..57767193, complement) | 4,860 | Vitamin D-dependent rickets type I |
| *CYP2R1* | NC_000011.10(14878005..14898915, complement) | 21,478 | Rickets due to defect in vitamin D 25-hydroxylation |
| *VDR* | NC_000012.12(47841537..47905031, complement) | 63,495 | Rickets, vitamin D-resistant, type IIA |
| *SLC34A3* | NC_000009.12(137230757..137236554) | 5,798 | Hypophosphatemic rickets with hypercalciuria |
| *FGF23* | NC_000012.12(4368227..4379728, complement) | 11,502 | Hypophosphatemic rickets, autosomal dominant |
| *PHEX* | NC_000023.11(157162075..157415286, complement) | 218,984 | Hypophosphatemic rickets, X-linked dominant |
| *EXT1* | NC_000008.11 (117799363..118111819, complement) | 314,324 | Exostoses, multiple, type 1 |
| *EXT2* | NC_000011.10(44095549..44245430) | 149,882 | Exostoses, multiple, type 1 |
| *ENPP1* | NC_000006.12(131808016..131895155) | 94,140 | Hypophosphatemic rickets, AR |
| *FAM20C* | NC_000007.14(192939..260774) | 72,410 | Hypophosphatemic rickets, AR |
| *IFT122* | NC_000003.12 (129440036..129520507) | 80,472 | Skull ectodermal dysplasia type 1 |
| *WDR35* | NC_000002.12 (19910260..19990123, complement) | 79,864 | Skull ectodermal dysplasia type 2 |
| *IFT43* | NC_000014.9 (75985753..76084073) | 98,321 | Skull ectodermal dysplasia type 3 |
| *WDR19* | NC_000004.12 (39182404..39285810) | 103,407 | Skull ectodermal dysplasia type 4 |
| *COL11A1* | NC_000001.11 (102876467..103108580, complement) | 232,114 | Fibrocartilage hyperplasia type 1 |
| *COL11A2* | NC_000006.12 (33162692..33193009, complement) | 30,318 | Fibrocartilage hyperplasia type 2 |
| *PRKAR1A* | NC_000017.11 (68413623..68551316) | 137,694 | Limb osteogenesis incomplete type 1 |
| *PDE4D* | NC_000005.10 (58969038..60524329, complement) | 1,519,061 | Limb osteogenesis incomplete type 2 |
| *ENPP1* | NC_000006.12 (131808016..131895155) | 87,140 | Hyponatremia rickets |
| *F8* | NC_000023.11(154835788..155022723, complement) | 186,936 | Hemophilia A |
| *F9* | NC_000023.11(139530720..139563459) | 32,740 | Hemophilia B |
| *F11* | NC_000004.12(186265964..186290727) | 24,783 | Factor XI deficiency |
| *VWF* | NC_000012.12(5948874..6124675, complement) | 175,802 | von Willebrand disease |
| *ZNF644* | NC_000001.11 (90915298..91022267, complement) | 106,972 | High myopia |
| *CCDC111* | NC_000004.12(184649613..184694959) | 45,347 | High myopia |
| *LEPREL1* | NC_000003.12 (189956728..190122437, complement) | 165,710 | High myopia |
| *LRPAP1* | NC_000004.12 (3503597..3532497, complement) | 28,901 | High myopia |
| *OPN1LW* | NC_000023.11 (154144224..154159032) | 14,809 | High myopia |
| *SCO2* | NC_000022.11 (50523568..50526439, complement) | 2,872 | High myopia |
| *SLC39A5* | NC_000012.12 (128395927..128401276, complement) | 7,811 | High myopia |
| *P4HA2* | NC_000005.10 (132190147..132227863, complement) | 37,717 | High myopia |
| *BSG* | NC_000019.10(571277..583493) | 12,217 | High myopia |
| *ARR3* | NC_000023.11(70268335..70281840) | 13,506 | High myopia |
| *SNTB1* | NC_000008.11(120535745..120812069, complement) | 276,325 | High myopia |
| *HLA-DQB1* | NC_000006.12(32659464..32666689, complement) | 7,226 | High myopia |
| *DSPP* | NC_000004.12 (87608529..87616873) | 8,345 | Dentin dysplasia |
| *RNU4ATAC* | NC_000002.12 (121530880..121531009) | 130 | MOPD type 1 |
| *PCNT* | NC_000021.9 (46324122..46445769) | 121,667 | MOPD type 2 |
| *ADAMTSL2* | NC_000009.12 (133532164..133575519) | 43,356 | Geleophysic dysplasia type 1 |
| *FBN1* | NC_000015.10 (48408306..48645788, complement) | 237,483 | Geleophysic dysplasia type 2 |
| *GH1* | NC_000017.11 (63917193..63918852, complement) | 1,660 | Growth hormone deficiency |
| *PKD1* | NC_000016.10 (2088708..2135898, complement) | 47,191 | Adult polycystic kidney disease |
| *PKD2* | NC_000004.12(88007647..88077779) | 70,133 | Adult polycystic kidney disease |
| *GANAB* | NC_000011.10 (62624826..62646726, complement) | 21,901 | Adult polycystic kidney disease |
| *PKHD1* | NC_000006.12 (51614685..52087625, complement) | 474,335 | Infantile polycystic kidney disease |

**Table S3. Functional prediction of the variants in *PHEX* and *SLC34A3***

|  | **Variant** | **PROVEN score** | **PolyPhen-2** |
| --- | --- | --- | --- |
| **Family 1** | *PHEX*: c.591A>G; p.(Gln197Gln) | 0 (Neutral) | NA |
| **Family 2** | *PHEX*: c.1661_1726del; p.(Glu554_Gly575del) | -82.358 (Deleterious) | NA |
| **Family 3** | *PHEX*: c.980A>G; p.(Tyr327Cys) / c.1078A>T; p.(Lys360*） | -1.207 (Neutral)/-6.902 (Deleterious) | 0.997 (Probably damaging)/ NA |
| **Family 4** | *PHEX*: c.1735G>A; p.(Gly579Arg) | -7.624 (Deleterious) | 1.000 (Probably damaging) |
| **Family 6** | *PHEX*: c.1017_1051dup; p.(Phe351Trpfs*16) | NA | NA |
| **Family 8** | *PHEX:* c.1699C>T; p. (Arg567*) | NA | NA |
| **Family 9** | SLC34A3: c.1336G>A; p.(Val446Ile）/c.1364T>C; p.(Leu455Pro) | 0.554 (Neutral)/-4.524 (Deleterious) | 0.998 (Probably damaging)/0.986  (Probably damaging) |
| **Splicing prediction** | | **Human Splicing Finder (version 3.1)** | |
| **Family 5** | *PHEX*: c.1079+1G>A; p.(?) | Alteration of the WT donor site, most probably affecting splicing | |
| **Family 7** | *PHEX:* c.1965+1G>A; p.(?) | Alteration of the WT donor site, most probably affecting splicing | |

**Figure S1**


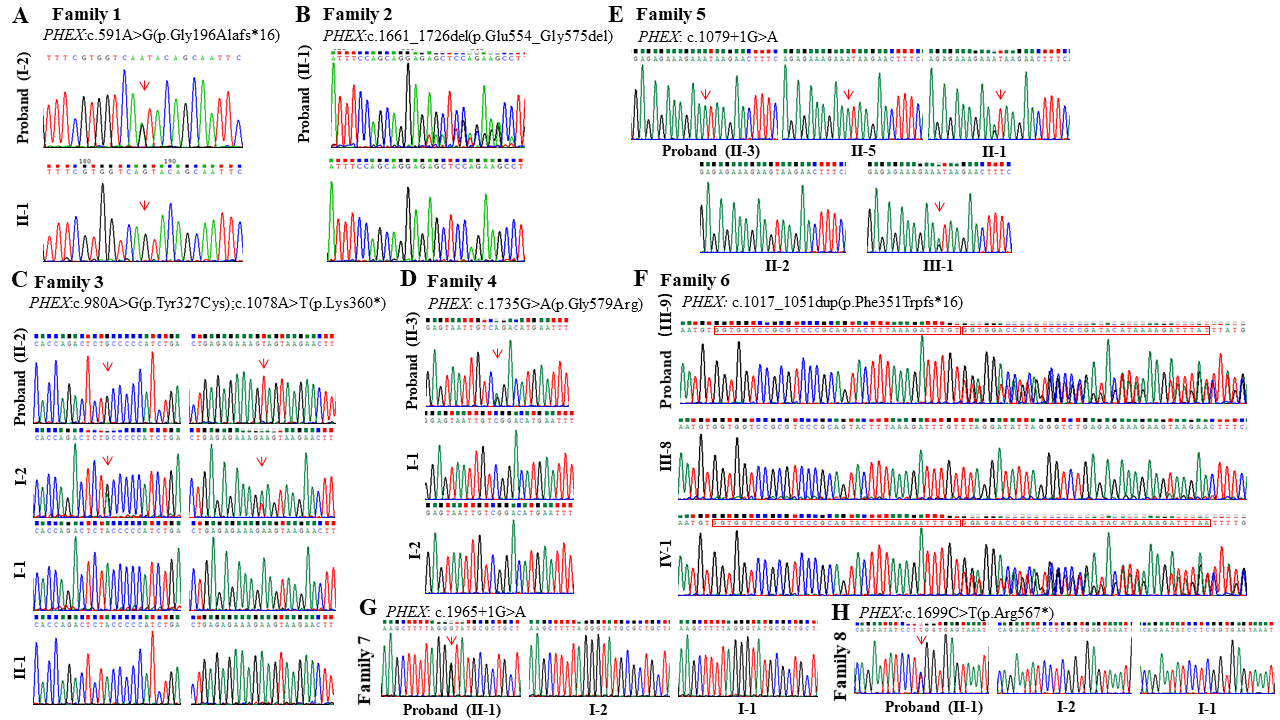


**Fig. S1 Sequencing analysis of individuals from Family 1-8 recruited in this study. A-H.** Variants confirmed in family 1 (A), family 2 (B), family 3 (C), family 4 (D), family 5 (E), family 6 (F), family 7 (G) and family 8 (H).


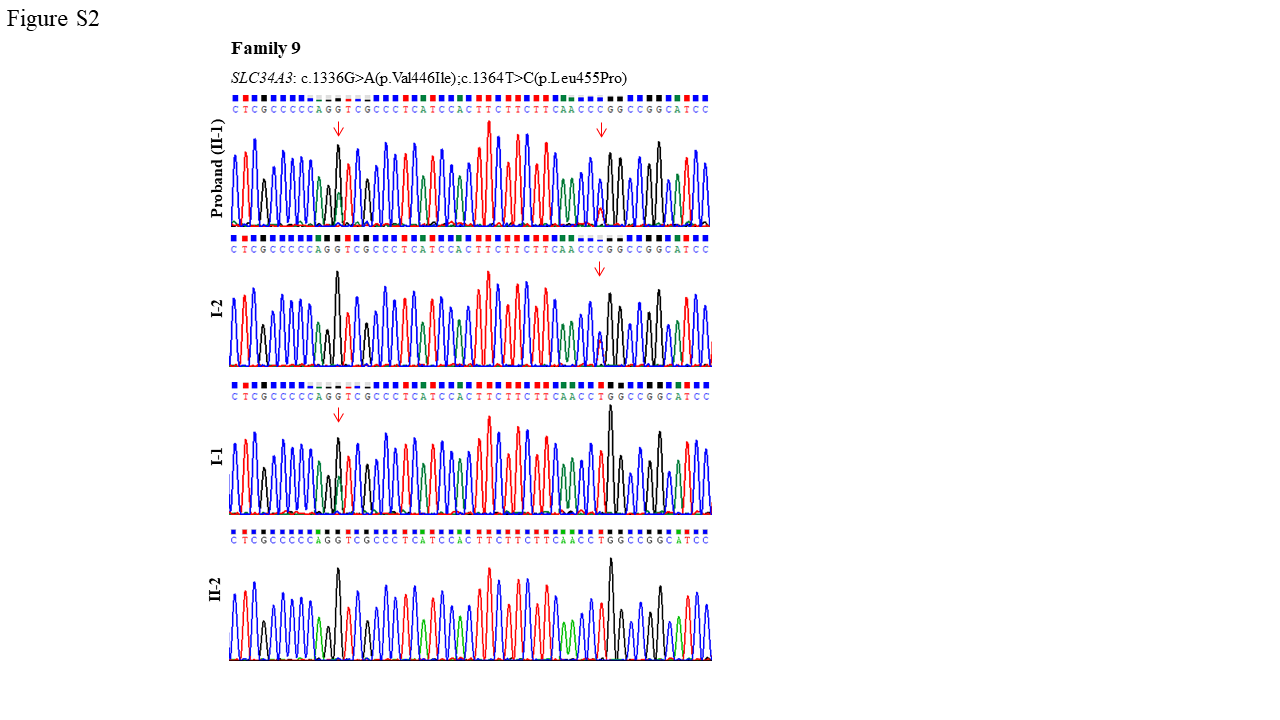


**Fig. S2 Sequencing analysis of individuals from Family 9.** The proband inherited the variant c.1336G>A (p.Val446Ile) from his father and inherited the variant c.1364T>C (p.Leu455Pro) from his mother, while his younger brother inherited both wildtype alleles.
